# Smoking behavior change and risk of cardiovascular disease incidence and mortality in patients with type 2 diabetes mellitus

**DOI:** 10.1186/s12933-023-01930-4

**Published:** 2023-07-29

**Authors:** Su-Min Jeong, Jung Eun Yoo, Junhee Park, Wonyoung Jung, Kyu Na Lee, Kyungdo Han, Cheol Min Lee, Ki-Woong Nam, Seung-Pyo Lee, Dong Wook Shin

**Affiliations:** 1 Department of Medicine, Seoul National University College of Medicine, Seoul, Republic of Korea; 2 Department of Family Medicine, Seoul National University Hospital, Seoul, Republic of Korea; 3 Department of Family Medicine, Seoul National University Health Service Center, Seoul, Republic of Korea; 4 Department of Family Medicine, Healthcare System Gangnam Center, Seoul National University Hospital, Seoul, Republic of Korea; 5 Department of Family Medicine, Samsung Medical Center, Sungkyunkwan University School of Medicine, Seoul, Republic of Korea; 6 Department of Family Medicine/Obesity and Metabolic Health Center, Kangdong Sacred Heart Hospital, Hallym University, Seoul, Republic of Korea; 7 Department of Statistics and Actuarial Science, Soongsil University, Seoul, Republic of Korea; 8 Department of Family Medicine, Seoul National University College of Medicine, Seoul, Republic of Korea; 9 Department of Neurology, Seoul National University College of Medicine and Seoul Metropolitan Government-Seoul National University Boramae Medical Center, Seoul, Republic of Korea; 10 Department of Internal Medicine and Cardiovascular Center, Seoul National University Hospital, Seoul, Republic of Korea; 11 Department of Clinical Research Design & Evaluation, Samsung Advanced Institute for Health Science & Technology (SAIHST), Sungkyunkwan University, 81 Irwon-Ro, Gangnam-gu, Seoul 06351 Republic of Korea

**Keywords:** Smoking cessation, Smoking reduction, Cardiovascular disease, Mortality, Diabetes mellitus

## Abstract

**Background:**

We aimed to examine the association between smoking behavior change and risk of cardiovascular disease (CVD) incidence and mortality in patients with type 2 diabetes mellitus (T2DM).

**Methods:**

This study used nationwide data from the Korean National Health Insurance System and included 349,137 T2DM patients who smoked. Smoking behavior changes were defined with five groups: quitters, reducers I (≥ 50% reduction), reducers II (20–50% reduction), sustainers (± 20%), and increasers (≥ 20% increase) from the number of cigarettes/day at the baseline.

**Results:**

During a median follow-up of 5.1 years, 6,514 cases of myocardial infarction (MI) (1.9%), 7,837 cases of ischemic stroke (IS) (2.2%), and 14,932 deaths (4.3%) were identified. Quitters had a significantly decreased risk of MI (adjusted hazard ratio [aHR] 0.80, 95% CI 0.75–0.86) and IS (aHR 0.80, 95% CI 0.75–0.85) compared to sustainers, whereas reducers did not have a significant association with the risk of MI (aHR 1.03, 95% CI 0.94–1.13) and IS (aHR 1.00, 95% CI 0.92–1.08) in reducer I. Quitters also had a lower all-cause and CVD mortality than sustainers.

**Conclusions:**

Smoking cessation was associated with decreased CVD incidence, and all-cause and CVD mortality among T2DM patients. However, smoking reduction was not associated with decreased risks for these.

**Supplementary Information:**

The online version contains supplementary material available at 10.1186/s12933-023-01930-4.

## Background

The global prevalence of type 2 diabetes mellitus (T2DM) was estimated to be 10.5% (536.6 million) in 2019 and was then predicted to continue to rise to 12.2% (783.2 million) in 2045 according to the International Diabetes Federation [1]. The increasing trend of T2DM prevalence indicates a major challenge to public health. Macrovascular (cardiovascular disease [CVD]) and microvascular (retinopathy, nephropathy, and diabetic neuropathy) complications can develop in people with T2DM. Moreover, the global mortality caused by diabetic vascular complication had increased, accounting for 26.8% of deaths among T2DM patients [2]. To reduce the development of its complications, management of modifiable lifestyle risk factor is imperative.

Cigarette smoking is a primary modifiable risk factor for developing T2DM complications. Cigarette smoking is well known to increase the risk of CVD incidence in the general population via various biological mechanisms such as endothelial dysfunction, activation of sympathetic nervous system, increased platelet activation, and thrombogenesis [3]. Moreover, risk of coronary heart disease and ischemic stroke in people with T2DM was more than twice as high as those without T2DM [4]. A meta-analysis demonstrated that cigarette smoking is significantly associated with increased risks of CVD events (relative risk [RR]: 1.44), all-cause mortality (RR: 1.55), CVD mortality (RR: 1.49) in patients with T2DM [5]. Furthermore, cigarette smoking accelerates risks of vascular complications in patients with T2DM [6, 7]. A Chinese cohort study reported bidirectional interaction between smoking and T2DM on CVD incidence, showing that T2DM patients were more susceptible to the detrimental effect of cigarette smoking than individuals without T2DM [8].

It is widely acknowledged that smoking cessation is associated with a reduced risk of CVD incidence [8–13], all-cause mortality [11, 13–15], and CVD mortality [11, 15] in patients with T2DM, as it is for the general population. However, almost all studies only focused on the effect of smoking cessation on CVD risk and mortality, which makes lack of evidence to understand how the smoking behavior change (e.g., reduced number of cigarette smoking per day) affect the risk of developing macrovascular complications and mortality in patients with T2DM, probably due to small number of studies [8–11, 13–15]. Unfortunately, smoking cessation all at once is not easy in real practice. In this context, smoking reduction could be an alternative step to mitigate the harmful effects of smoking [16]. Studies about health benefits from smoking reduction without cessation showed mixed results in the general population. A recent meta-analysis reported that smoking reduction was related to decreased risk of lung cancer, but not for CVD incidence or all-cause mortality [17]. Only one study of Korean men with T2DM suggested the possible benefit of smoking reduction for CVD risk, and that was without statistical significance, thus suggesting the necessity of further evaluation [13]. However, that study did not consider baseline smoking intensity or T2DM status, which is an important confounding factor for developing macrovascular complication or mortality.

Therefore, our study examined the association between smoking behavior change, in particular smoking reduction, and the risk of CVD incidence and all-cause/CVD mortality among patients with T2DM. In addition, we performed stratified analysis to evaluate this association by T2DM status.

## Methods

### Data source and study setting

Our study’s data came from the database provided by the National Health Insurance System (NHIS) in Korea, which is a single insurer and provides mandatory universal insurance covering about 97% of the whole population. The remaining 3% of the population with low income is covered by the Medical Aid Program. The NHIS recommends that all insured individuals over 40 years of age and all employees regardless of age have a general health screening at least every 2 years [18]. This national health screening consists of a standard questionnaire (regarding past medical history, current medications, and lifestyle habits, such as drinking, smoking, and exercise), anthropometric measurements (height, weight, body mass index, and blood pressure), and laboratory tests. In addition, it could be linked with the information on claimed health care usage, which has been widely employed by epidemiological studies. Hence, the NHIS retains an extensive health information dataset of the entire Korean population.

### Study population

**Fig. 1:**
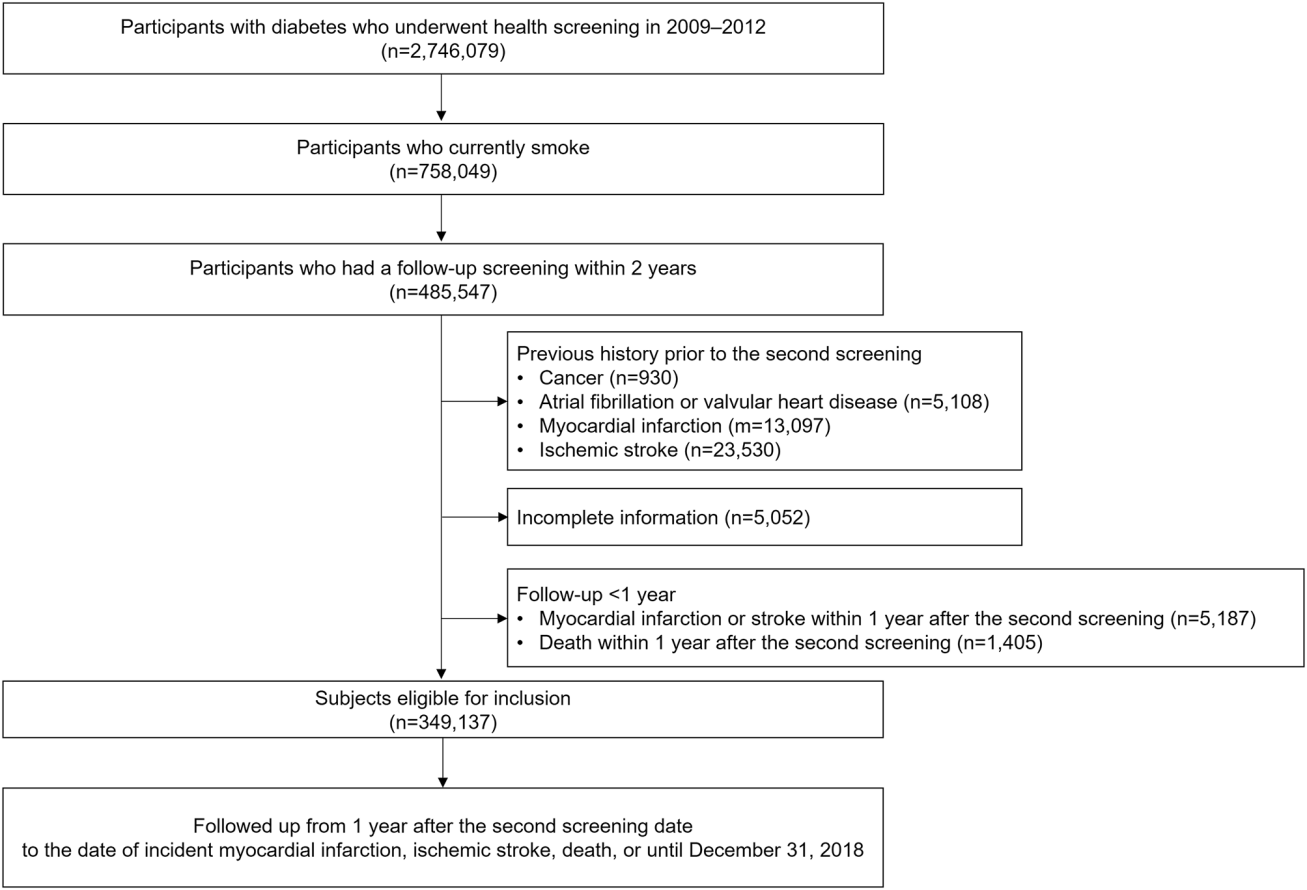
Flow chart of the study population

Among individuals who underwent a health screening in the period of 2009–2012, we identified 2,746,079 individuals with T2DM: [1] those with fasting plasma glucose ≥ 126 mg/dL at the health screening and [2] those with a previous history of T2DM who had evidence of at least one claim per year for prescription of antidiabetic agents with International Classification of Disease (ICD)-10 code (E11–14) before the health screening. This definition was based on the consensus of relevant findings widely used in previous studies [19, 20]. We then selected current smokers (n = 758,049) according to the definition of current smokers by World Health Organization [21]. Among them, 485,547 participants underwent a follow-up health examination within 2 years. We excluded those who had previously been diagnosed with any cancer (n = 54,192), atrial fibrillation, or valvular heart diseases (n = 5,108), myocardial infarction (MI; n = 13,097), or ischemic stroke (n = 23,530) before the second health screening. This was to eliminate baseline characteristics that could influence the primary outcomes. Those with missing variable used in the study were also excluded (n = 33,891). To reduce the effect of reverse causality, we applied a 1-year lag time by excluding participants who were diagnosed with MI or ischemic stroke (n = 5,187) and who died (n = 1,405) within 1 year after the second health examination period. Finally, we included a total of 349,137 participants (Fig. 1).

This study was approved by the Institutional Review Board of Samsung Medical Center (IRB File No. SMC 2022-07-072).

### Definition of change in amount of smoking cigarettes

Information on smoking status and smoking behavior change was obtained from a self-administered questionnaire of national health examination in the NHIS. The participants who answered that they have smoked ≥100 cigarettes in his or her lifetime and currently smoke cigarettes were then asked about their average number of cigarettes per day and their duration of smoking in years. According to the number of cigarettes in the first examination, participants were categorized into three groups: [1] light smokers (< 10 cigarettes per day), [2] moderate smokers (10–19 cigarettes per day), and [3] heavy smokers (≥ 20 cigarettes per day). Then, study participants in each of the three groups were recategorized into five subgroups based on the difference in the number of cigarettes smoked per day between the first examination and follow-up examination: quitter, reducer I, reducer II, sustainer, and increaser. These five subgroups were defined as follows: [1] quitters were those who had quit smoking, [2] reducers I were those who reduced the number of cigarettes by 50% or more, [3] reducers II were those who reduced the number of cigarettes by 20% or more and less than 50%, [4] sustainers were those who reduced the number of cigarettes by less than 20% or increased by less than 20%, and [5] increasers were those who increased the number of cigarettes by 20% or more.

### Covariates

The demographic variables including age, sex, and area of residence were considered as potential covariates. Household income was categorized into quartiles based on insurance premium levels (in Korea, insurance premiums are determined by income level), with those covered by Medical Aid (the 3% poorest) merged into the lowest income quartile. Drinking was classified into the following four levels according to the amount of alcohol consumption per day: [1] none, [2] mild (< 15 g of alcohol/day), [3] moderate (15–30 g of alcohol/day), and [4] heavy (≥ 30 g/day). Regular physical activity was defined as moderate physical activity for more than 30 min and more than 5 days per week during the past week. Body mass index was calculated using weight (kg) divided by height in meters squared (m^2^). Comorbidities were defined as having both ICD-10 codes (for hypertension, ICD-10 codes of I10-I13 or I15; for dyslipidemia, ICD-10 codes of E78; for chronic kidney disease, ICD-10 codes of N18 or N19; and for chronic pulmonary obstructive disease, ICD-10 codes of J41-J44) and prescription of relevant medication.

T2DM patients were categorized considering T2DM status; the duration of T2DM (new-onset T2DM, T2DM duration < 5 years, and T2DM duration ≥ 5 years), the number of antidiabetic agents used (0, 1–2, and ≥ 3), and use of insulin.

### Study outcomes and follow-up

Primary endpoints of this study are newly diagnosed MI, ischemic stroke, and death. Newly diagnosed MI and ischemic stroke were identified on the basis of the ICD-10 code for MI (ICD-10 codes I21 or I22 during hospitalization or these codes’ having been recorded at least two times for outpatients) and ischemic stroke (ICD-10 codes I63 or I64 during hospitalization with claims for brain magnetic resonance imaging [MRI] or brain computerized tomography [CT]), respectively. Mortality data were obtained from Statics Korea including the cause of death MI mortality (I21-22) and ischemic stroke mortality (I63-64). The cohort was followed from 1 year after the second health examination date to the date of incident MI, ischemic stroke, death, or until the end of the study period (December 31, 2018), whichever came first.

### Statistical analysis

Continuous variables were presented as means ± standard deviation (SD), and categorical variables were presented as numbers and percentages. The incidence rates of MI, ischemic stroke, and death are presented per 1,000 person-years. Cox proportional hazards regression analysis was conducted to evaluate the association between smoking behavior change and the incident of MI, ischemic stroke, and death among individuals with T2DM.

The final model was adjusted for age, sex, income, area of residence, alcohol consumption, duration of smoking, regular physical activity, body mass index, comorbidities (hypertension, dyslipidemia, chronic kidney disease, and chronic pulmonary obstructive disease), fasting glucose level, the number of oral antidiabetic agents used, and insulin use. Stratification analyses by smoking levels at the first examination (light, moderate, and heavy smoker), age (< 65 years and ≥ 65 years) and sex were performed to see the different association of smoking behavior change with incidence of MI, ischemic stroke, and death. Statistical analyses were performed using SAS version 9.4 (SAS Institute Inc., Cary, NC, USA), and a *P* value < 0.05 was considered statistically significant.

## Results

### Baseline characteristics of study participants

Table 1 shows baseline characteristics in the period of the second examination according to the smoking behavior change (quitters, reducers I, reducers II, sustainers, and increasers). The mean age of the total study participants was 51.6 years, and 95.2% of the participants were men. In this study population, 9.3% were light smokers, 36.4% were moderate smokers, and 54.3% were heavy smokers in the first examination. Of note, 20.7% (7.6% and 13.1% in reducer I and reducer II, respectively) and 18.2% of total participants reduced or quit smoking during a 2-year interval, respectively, whereas 16.3% increased their smoking amount. The quitters were more likely to be light smokers with short duration of smoking, whereas reducers I were more likely to be heavy smokers with a longer duration of smoking. The quitters and reducers I tended to have high prevalence of comorbidities and T2DM for 5 years or more with multiple antidiabetic agents and insulin than the group of sustainers.

**Table 1:** Baseline characteristics of the study population

Variables	Total (N = 349,137)	Smoking behavior change					
		Quitter (N = 63,694)	Reducer I (N = 26,516)	Reducer II (N = 45,832)	Sustainer (N = 156,329)	Increaser (N = 56,766)	P value
Age (years)	51.6 ± 10.9	54.0 ± 11.1	52.9 ± 11.5	50.7 ± 10.8	51.1 ± 10.6	50.8 ± 11.0	< 0.001
Sex (men)	332,461 (95.2)	58,025 (91.1)	24,828 (93.6)	44,289 (96.6)	151,401 (96.8)	53,918 (95.0)	< 0.001
Income							< 0.001
Q1(lowest)	63,355 (18.2)	11,556(18.1)	5,567 (21.0)	8,054 (17.6)	27,633 (17.7)	10,545 (18.6)	
Q2	67,593 (19.4)	12,150(19.1)	5,472 (20.6)	8,843 (19.3)	29,613 (18.9)	11,515 (20.3)	
Q3	103,559 (29.7)	17,453(27.4)	7,493 (28.3)	13,890 (30.3)	47,481 (30.4)	17,242 (30.4)	
Q4(highest)	114,630 (32.8)	22,535(35.4)	7,984 (30.1)	15,045 (32.8)	51,602 (33.0)	17,464 (30.8)	
Alcohol consumption							< 0.001
Non	97,091 (27.8)	25,781 (40.5)	7,729 (29.2)	11,307 (24.7)	38,030 (24.3)	14,244 (25.1)	
Mild	111,621 (32.0)	19,429 (30.5)	9,931 (37.5)	15,773 (34.4)	49,237 (31.5)	17,251 (30.4)	
Moderate	77,095 (22.1)	10,428 (16.4)	5,352 (20.2)	10,686 (23.3)	37,757 (24.2)	12,872 (22.7)	
Heavy	63,330 (18.1)	8,056 (12.7)	3,504 (13.2)	8,066 (17.6)	31,305 (20.0)	12,399 (21.8)	
Smoking status							< 0.001
Light, < 10 cigarettes/day	32,551 (9.3)	11,463 (18.0)	1,057 (4.0)	2,348 (5.1)	5,561 (3.7)	12,122 (21.4)	
Moderate, 10–19 cigarettes/day	127,032 (36.4)	24,789 (38.9)	5,276 (19.9)	15,046 (32.8)	50,140 (32.1)	31,781 (56.0)	
Heavy, ≥ 20 cigarettes/day	189,554 (54.3)	27,442 (43.1)	20,183 (76.1)	28,438 (62.1)	100,628 (64.4)	12,863 (22.7)	
Duration of smoking (years)							< 0.001
<5	10,090 (2.9)	3,575 (5.6)	655 (2.47)	845 (1.8)	2,790 (1.8)	2,225 (3.9)	
5–9y	13,828 (4.0)	3,499 (5.5)	1,101 (4.15)	1,518 (3.3)	4,862 (3.1)	2,848 (5.0)	
10–19	81,273 (23.3)	14,165 (22.2)	5,780 (21.8)	10,864 (23.7)	35,359 (22.6)	15,105 (26.6)	
20–29	116,486 (33.4)	18,894 (29.7)	8,205 (30.94)	15,788 (34.5)	55,080 (35.2)	18,519 (32.6)	
≥30	127,460 (36.5)	23,561 (37.0)	10,775 (40.64)	16,817 (36.7)	58,238 (37.3)	18,069 (31.8)	
Pack-years of smoking							< 0.001
<10	74,821 (21.4)	19,913 (31.3)	3,738 (14.1)	6,555 (14.3)	23,688 (15.2)	20,927 (36.9)	
10–20	97,025 (27.8)	16,790 (26.4)	5,925 (22.3)	11,924 (26.0)	42,754 (27.4)	19,632 (34.6)	
20–30	79,295 (22.7)	12,053 (18.9)	6,090 (23.0)	10,169 (22.2)	42,092 (26.9)	8,891 (15.7)	
≥30	97,996 (28.1)	14,938 (23.5)	10,763 (40.6)	17,184 (37.5)	47,795 (30.6)	7,316 (12.9)	
Physical activity							< 0.001
Non	277,155 (79.4)	48,323 (75.9)	20,600 (77.7)	36,399 (79.4)	125,883 (80.5)	45,950 (81.0)	
Irregular	53,160 (15.2)	11,140 (17.5)	4,307 (16.2)	7,124 (15.5)	22,601 (14.5)	7,988 (14.1)	
Regular	18,822 (5.4)	4231 (6.6)	1,609 (6.1)	2,309 (5.0)	7,845 (5.0)	2,828 (5.0)	
Body mass index (kg/m^2^)	24.8 ± 3.3	25.0 ± 3.2	24.7 ± 3.3	24.8 ± 3.4	24.8 ± 3.3	24.8 ± 3.4	< 0.001
Systolic blood pressure (mmHg)	126.8 ± 14.5	127.2 ± 14.5	127.0 ± 14.9	126.7 ± 14.4	126.7 ± 14.4	126.7 ± 14.7	< 0.001
Diastolic blood pressure (mmHg)	79.0 ± 9.9	78.8 ± 9.8	78.9 ± 9.9	79.1 ± 9.9	79.1 ± 9.9	79.0 ± 10.0	< 0.001
Fasting glucose (mg/dL)	137.8 ± 51.8	138.6 ± 50.8	137.9 ± 52.6	136.6 ± 50.8	137.4 ± 51.6	139.1 ± 54.0	< 0.001
Total cholesterol (mg/dL)	194.0 ± 43.3	192.9 ± 46.0	192.9 ± 47.0	193.9 ± 41.5	194.5 ± 42.6	194.1 ± 41.5	< 0.001
Estimated glomerular filtration rate	92.8 ± 48.6	90.7 ± 48.9	92.2 ± 46.1	93.3 ± 48.2	93.3 ± 48.7	93.7 ± 49.5	< 0.001
Comorbidity							
Hypertension	157,714 (45.2)	31,276 (49.1)	12,481 (47.1)	20,172 (44.0)	69,010 (44.1)	24,775 (43.6)	< 0.001
Dyslipidemia	129,888 (37.2)	26,053 (40.9)	9,845 (37.1)	16,690 (36.4)	56,738 (36.3)	20,562 (36.2)	< 0.001
Chronic kidney disease	15,585 (4.5)	3,824 (6.0)	1,410 (5.3)	1,874 (4.1)	6,145 (3.9)	2,332 (4.1)	< 0.001
COPD	87,696 (25.1)	18,162 (28.5)	7,116 (26.8)	10,902 (23.8)	37,576 (24.0)	13,940 (24.6)	< 0.001
Diabetes duration							< 0.001
New-onset	198,800 (56.9)	32,857 (51.6)	14,553 (54.9)	27,078 (59.1)	91,560 (58.6)	32,752 (57.7)	
<5 years	79,605 (22.8)	15,506 (24.3)	6,254 (23.6)	9,965 (21.7)	34,867 (22.3)	13,013 (22.9)	
≥5 years	70,732 (20.3)	15,331 (24.1)	5,709 (21.5)	8,789 (19.2)	29,902 (19.1)	11,001 (19.4)	
Number of antidiabetic agents used							< 0.001
0	172,956 (49.5)	27,042 (42.5)	12,671 (47.8)	23,830 (52.0)	80,418 (51.4)	28,995 (51.1)	
1–2	124,456 (35.7)	25,584 (40.2)	9,693 (36.6)	15,646 (34.1)	53,989 (34.5)	19,544 (34.4)	
≥3	51,725 (14.8)	11,068 (17.4)	4,152 (15.7)	6,356 (13.9)	21,922 (14.0)	8,227 (14.5)	
Use of insulin	17,474 (5.0)	4,227 (6.6)	1,436 (5.4)	2,105 (4.6)	6,960 (4.5)	2,746 (4.8)	< 0.001

Data are presented as number (%) or mean ± standard deviation

COPD, chronic obstructive pulmonary disease

### The risk of CVD and death according to smoking behavior change among individuals with T2DM

There were 6,514 cases of MI (1.9%), 7,837 cases of ischemic stroke (2.2%), and 14,932 deaths (4.3%) during median follow-up of 5.1 years. Quitters had a significantly decreased risk of MI (aHR 0.80, 95% CI 0.75–0.86) and ischemic stroke (aHR 0.80, 95% CI 0.75–0.85) compared to sustainers. The absolute risk difference among quitters was − 0.38 (-0.49, -0.27) for MI, corresponding to a number needed to treat (NNT) of about 2.6. For ischemic stroke, the absolute risk difference was − 0.46 (-0.59, -0.33), with an NNT of about 2.2. Reducers did not have a significant association with the risk of MI (aHR 1.03, 95% CI 0.94–1.13 in reducer I) and ischemic stroke (aHR 1.00, 95% CI 0.92–1.08 in reducer I) compared to sustainers (Table 2).

**Table 2:** Hazard ratios and 95% confidence intervals for the incidence of myocardial infarction, ischemic stroke, and mortality according to smoking behavior change

Smoking behavior change	Event (n)	Duration (person-years)	IR	Hazard ratio (95% confidence interval)			5-year absolute risk (%) (95% CI)	Risk difference (95% CI)	No. Needed to Treat
				Model 1	Model 2	Model 3			
Cardiovascular disease events									
Myocardial infarction									
Quitter	1,171	314,614.7	3.7	1.00 (0.93–1.07)	**0.81 (0.76–0.87)**	**0.80 (0.75–0.86)**	1.54 (1.45, 1.63)	**-0.38 (-0.49, -0.27)**	2.6
Reducer I	573	129,894.0	4.4	**1.19 (1.08–1.30)**	1.04 (0.95–1.14)	1.03 (0.94–1.13)	1.98 (1.81, 2.14)	0.06 (-0.12, 0.24)	16.7
Reducer II	823	226,066.8	3.6	0.98 (0.91–1.06)	0.99 (0.92–1.07)	0.99 (0.92–1.07)	1.90 (1.77, 2.03)	-0.02 (-0.16, 0.12)	50
Sustainer	2,856	768,275.4	3.7	1 (Ref.)	1 (Ref.)	1 (Ref.)	1.92 (1.84, 1.99)	Ref.	Ref.
Increaser	1,091	277,276.4	3.9	1.06 (0.99–1.14)	1.06 (0.99–1.14)	1.05 (0.98–1.13)	2.02 (1.90, 2.14)	0.10 (-0.03, 0.23)	10
P value				0.001	< 0.001	< 0.001			
Ischemic stroke									
Quitter	1,420	313,782.0	4.5	1.00 (0.94–1.06)	**0.82 (0.77–0.87)**	**0.80 (0.75–0.85)**	1.90 (1.80, 2.00)	**-0.46 (-0.59, -0.33)**	2.2
Reducer I	699	129,406.0	5.4	**1.19 (1.10–1.30)**	1.01 (0.93–1.09)	1.00 (0.92–1.08)	2.35 (2.18, 2.52)	-0.01 (-0.20, 0.18)	100
Reducer II	948	225,652.8	4.2	0.93 (0.86–1.00)	0.95 (0.89–1.02)	0.95 (0.89–1.02)	2.25 (2.11, 2.39)	-0.11 (-0.27, 0.05)	9.1
Sustainer	3,466	766,293.8	4.5	1 (Ref.)	1 (Ref.)	1 (Ref.)	2.36 (2.28, 2.44)	Ref.	Ref.
Increaser	1,304	276,608.2	4.7	1.04 (0.98–1.11)	1.05 (0.98–1.11)	1.04 (0.97–1.10)	2.44 (2.31, 2.57)	0.08 (-0.07, 0.23)	12.5
P value				< 0.001	< 0.001	< 0.001			
**Mortality**									
All-cause mortality									
Quitter	2,881	316,956.8	9.1	**1.11 (1.07–1.16)**	**0.92 (0.88–0.96)**	**0.90 (0.86–0.94)**	3.63 (3.50, 3.77)	**-0.40 (-0.56, -0.24)**	2.5
Reducer I	1,520	131,023.4	11.6	**1.43 (1.35–1.51)**	**1.15 (1.09–1.22)**	**1.14 (1.08–1.21)**	4.55 (4.34, 4.77)	0.52 (0.29, 0.75)	2.0
Reducer II	1,832	227,806.9	8.0	0.99 (0.94–1.04)	1.02 (0.97–1.07)	1.02 (0.97–1.07)	4.10 (3.93, 4.28)	0.07 (-0.13, 0.27)	14.3
Sustainer	6,294	774,303.1	8.1	1 (Ref.)	1 (Ref.)	1 (Ref.)	4.03 (3.94, 4.13)	Ref.	Ref.
Increaser	2,405	279,628.3	8.6	**1.06 (1.01–1.11)**	**1.07 (1.02–1.12)**	**1.06 (1.01–1.11)**	4.24 (4.08, 4.40)	0.21 (0.02, 0.40)	4.8
P value				< 0.001	< 0.001	< 0.001			
Myocardial infarction mortality									
Quitter	122	316,956.8	0.4	1.08 (0.87–1.33)	0.81 (0.65–1.00)	**0.79 (0.64–0.98)**	0.15 (0.12, 0.18)	**-0.04 (-0.08, 0.00)**	25
Reducer I	69	131,023.4	0.5	**1.48 (1.13–1.92)**	1.18 (0.90–1.54)	1.16 (0.89–1.51)	0.22 (0.17, 0.27)	0.03 (-0.03, 0.09)	33.3
Reducer II	76	227,806.9	0.3	0.94 (0.73–1.21)	0.94 (0.73–1.21)	0.94 (0.73–1.21)	0.18 (0.14, 0.22)	-0.01 (-0.06, 0.04)	100
Sustainer	276	774,303.1	0.4	1 (Ref.)	1 (Ref.)	1 (Ref.)	0.19 (0.17, 0.21)	Ref.	Ref.
Increaser	104	279,628.3	0.4	1.05 (0.83–1.31)	1.06 (0.84–1.33)	1.05 (0.84–1.32)	0.20 (0.16, 0.24)	0.01 (-0.04, 0.06)	100
P value				0.046	0.099	0.084			
Ischemic stroke mortality									
Quitter	46	316,956.8	0.1	1.04 (0.73–1.46)	**0.69 (0.48–0.98)**	**0.67 (0.47–0.95)**	0.05 (0.04, 0.07)	**-0.03 (-0.05, -0.01)**	33.3
Reducer I	25	131,023.4	0.2	1.37 (0.88–2.11)	0.91 (0.59–1.41)	0.89 (0.58–1.39)	0.07 (0.04, 0.10)	-0.01 (-0.04, 0.02)	100
Reducer II	31	227,806.9	0.1	0.97 (0.65–1.45)	0.98 (0.65–1.46)	0.98 (0.66–1.46)	0.08 (0.05, 0.11)	0.00 (-0.03, 0.03)	NA
Sustainer	108	774,303.1	0.1	1 (Ref.)	1 (Ref.)	1 (Ref.)	0.08 (0.06, 0.10)	Ref.	Ref.
Increaser	43	279,628.3	0.2	1.11 (0.78–1.57)	1.02 (0.71–1.45)	1.01 (0.71–1.44)	0.08 (0.06, 0.10)	0.00 (-0.03, 0.03)	NA
P value				0.684	0.291	0.222			

IR, incidence rate per 1,000 person-years

Model 1: Unadjusted

Mode 2: Adjusted for age, sex, income, area of residence, alcohol consumption, duration of smoking, physical activity, body mass index, and comorbidities (hypertension, dyslipidemia, chronic kidney disease, and chronic obstructive pulmonary disease)

Mode 3: Model 2 + adjusted for fasting glucose, duration of diabetes, and use of insulin

Regarding mortality, quitters also had a lower all-cause mortality (aHR 0.90, 95% CI 0.86–0.94), MI mortality (aHR 0.79, 95% CI 0.64–0.98), and ischemic stroke mortality (aHR 0.67, 95% CI 0.47–0.95) than sustainers. Reducers had an increased risk of all-cause mortality (aHR 1.14, 95% CI 1.08–1.21 in reducer I) and did not have a significant association with MI mortality (aHR 1.16, 95% CI 0.89–1.51 in reducer I) and ischemic stroke mortality (aHR 0.89, 95% CI 0.58–1.39 in reducer I) compared to sustainers. Increasers showed increased all-cause mortality compared with sustainers (aHR 1.06, 95% CI 1.01–1.11) (Table 2).

**Fig. 2:**
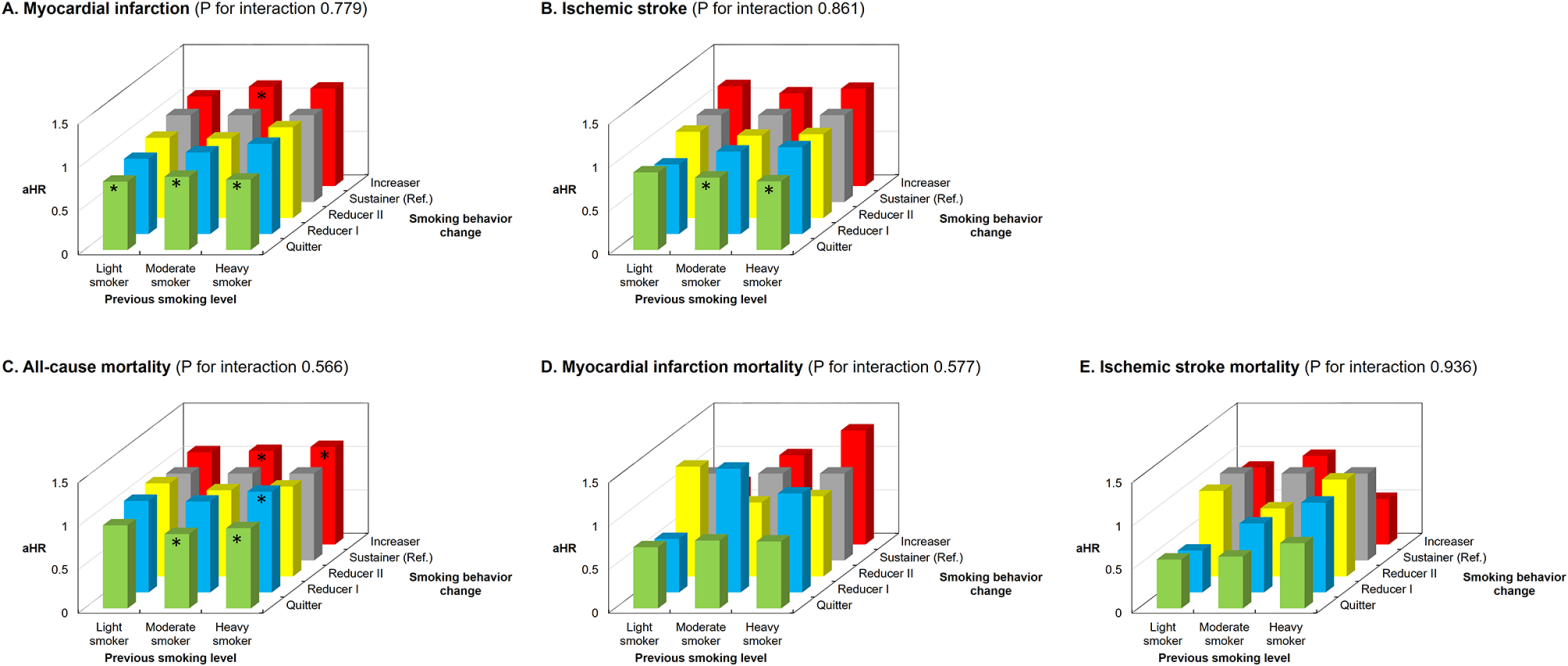
The association of smoking behavior change with myocardial infarction, ischemic stroke, and mortality stratified by previous smoking level. **(A)** Myocardial infarction; **(B)** Ischemic stroke; **(C)** All-cause mortality; **(D)** Myocardial infarction mortality; **(E)** Ischemic stroke mortality. Adjusted for age, sex, income, area of residence, alcohol consumption, duration of smoking, physical activity, body mass index, comorbidities (hypertension, dyslipidemia, chronic kidney disease, and chronic obstructive pulmonary disease), fasting glucose, duration of diabetes, and use of insulin. aHR, adjusted hazard ratio; *P < 0.05

A similar trend was noted in analysis stratified by smoking level at the first examination (light, moderate, and heavy smokers) (Fig. 2). The association in light smokers was less significant than that in moderate and heavy smokers (Supplementary Table S1).

### Stratified analysis by diabetes mellitus status

**Fig. 3:**
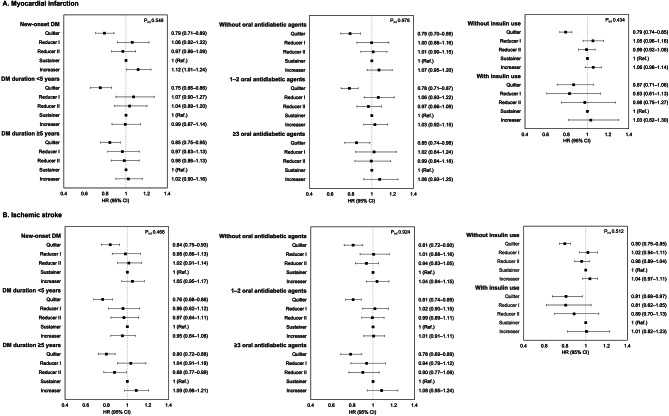
Association of smoking behavior change on the incidence of myocardial infarction and ischemic stroke according to type 2 diabetes mellitus status. (**A)** Myocardial infarction; **(B)** Ischemic stroke. Adjusted for age, sex, income, area of residence, alcohol consumption, duration of smoking, physical activity, body mass index, comorbidities (hypertension, dyslipidemia, chronic kidney disease, and chronic obstructive pulmonary disease), fasting glucose, duration of diabetes, and use of insulin. HR, hazard ratio; CI, confidence interval

The associations between smoking behavior change and CVD incidence according to duration of T2DM, number of oral antidiabetic agents, and use of insulin are shown in Fig. 3. The overall effects of smoking behavior change on CVD incidence were consistent across the T2DM status (*P* for interaction ≥ 0.05). Quitters had 13–25% reduced risk of MI and 16–24% reduced risk of ischemic stroke. Of note, the risk of MI incidence was reduced among those who quitted smoking (aHR 0.79, 95% CI 0.71–0.89) and increased among increasers (aHR 1.12, 95% CI 1.01–1.24) among patients with new-onset T2DM.

All-cause mortality was consistently lower in quitters than in sustainers regardless of T2DM status. Although similar trends were observed for MI mortality and stroke mortality, the statistical significance of the results was not found (Supplementary Figure S1).

### Stratified analysis by age and sex

In stratified analyses according to age and sex, the results were generally consistent with the main findings (Supplementary Table S2 and S3). The association between smoking behavior change and all-cause mortality was more prominent for the young aged group (40–64 years, *P* for interaction < 0.036). In addition, more significant association was shown in men than women, despite nonsignificant *P* values for interaction by sex.

## Discussion

Our study demonstrated that smoking cessation in patients with T2DM is associated with a 20% lower risk of MI and ischemic stroke incidence, 10% lower risk of all-cause mortality, and 21% MI and 34% ischemic stroke mortality compared to sustaining smoking intensity ( < ± 20% of change in number of cigarettes per day from baseline). However, smoking reduction was not significantly associated with a decreased risk of CVD incidence or all-cause/CVD mortality. These findings did not differ by severity of T2DM status. To our best knowledge, none of previous studies examined how smoking behavior change including smoking reduction is associated with CVD incidence or mortality in patients with T2DM considering the baseline smoking intensity and T2DM status. The use of large national population is a key strength in our study, allowing us to verify risk of CVD and mortality according to smoking behavior change.

Smoking cessation is major lifestyle modification for CVD risk reduction among patients with T2DM. The health benefits from smoking cessation are undoubtedly acknowledged. Our study also found that smoking cessation might have a 20% reduced risk of CVD incidence among patients with T2DM consistent with previous studies [11, 15]. The potential mechanisms of reduced CVD risk and mortality related to smoking cessation can be adopted by reducing harmful effect of smoking. Cigarette smoking causes impaired endothelium-dependent vasodilation and inflammatory response promoting atherosclerosis as well as platelet aggregation and prothrombotic process leading to thrombosis [22].

A few studies have examined the association between smoking cessation and all-cause mortality and CVD mortality among T2DM patients suggesting a reduced mortality related to smoking cessation [11, 13–15]. Cigarette smoking contributes mortality from any causes including CVD, cancer, and chronic obstructive pulmonary disease in the general population, which is expected to be similar in T2DM patients [23]. Among T2DM patients, cigarette smoking may accelerate CVD mortality, showing a substantial interaction with T2DM [24]. Cigarette smoking is independently responsible for micro/macrovascular T2DM complications [25] as well as deteriorated glucose homeostasis (e.g., increased insulin resistance) [26]. Nicotine, a major component of cigarette smoking, is associated with skeletal muscle insulin resistance via sympathetic nervous system activation, increased mammalian target of rapamycin (mTOR)/p70S6K activity [27] and increased delivery of free fatty acid to the liver favoring fat accumulation, [28] suggesting additional negative effects of cigarette smoking on glucose metabolism.

However, reduced all-cause mortality from smoking cessation was estimated to be 22–50% in the literature (aHR ranging from 0.51 to 0.78), which is more pronounced than the 10% reduced all-cause mortality in our study (aHR 0.90 of all-cause mortality). This discrepancy might be due to a difference in duration since the smoking cessation. The duration of smoking cessation in our study is expected to be 1–3 years shorter than in other studies (e.g., 6 years; [11] and longer duration since smoking cessation could bring greater risk reduction [29]. Our findings thus added evidence that mortality could be decreased even a few years after quitting. In stratified analysis by age, reduced mortality related to smoking cessation was more pronounced in the young age group (< 65 years) compared to a counterpart consistent with prior findings [30]. Less significance in the older age group might be due to high absolute mortality in older age among both quitters and sustainers that attenuated the relative risk difference.

Our findings do not support that smoking reduction mitigates the harmful effects of cigarette smoking for CVD incidence, all-cause mortality, or CVD mortality, which is similar to the results in general population [31, 32]. Our prior study in the general population alluded to a low threshold effect that low-level smoking could cause a substantial risk for CVD incidence, suggesting that there is no safe level of smoking [33]. For instance, even low-level smoking intensity could bring maximal harmful effects of cigarette smoking such as platelet aggregation [34]. Meanwhile, reducers I unexpectedly had an increased risk of all-cause mortality in our study (aHR 1.14). There could be a residual confounding effect, even though we adjusted for duration of smoking and comorbidities. Reducers (especially reducer I) in our study tended to be heavy smokers with a longer duration of smoking than other groups. The results from stratified analysis by baseline smoking intensity showed an increased risk of all-cause mortality in reducers only among heavy smokers. In addition, the sick quitter (reducer) effect might interfere with the results in part. Ill patients tend to profoundly limit their smoking amount, which affects mortality, and reducer I in our study had a higher proportion of comorbidities than the other groups.

When indirectly compared to the results from our previous study on the general population, the NNT for quitting cigarettes to reduce the incidence of myocardial infarction (MI) and ischemic stroke was found to be lower [31]. In addition, the association of smoking behavior change with CVD risk and mortality was not changed by T2DM status, however, it is noteworthy that the absolute risk reduction from smoking cessation would be greater in severe T2DM patients (e.g., long duration of T2DM and insulin use), given a higher risk of CVD incidence [35] and mortality [36] as the duration of T2DM increased. The current guidelines from the American Diabetes Association [37] and European Society of Cardiology [38] clinically advocate both not to use cigarettes and to provide smoking cessation counseling and treatment in all T2DM patients who smoke cigarettes. In a clinical point of view, the time when T2DM was newly diagnosed could be a teachable moment to improve their lifestyle habits including smoking cessation [39]. However, 34.5% of patients remained as current smokers even after a new diagnosis of T2DM [15]. Our study also found that only 16.5% of patients newly diagnosed with T2DM quit smoking. Therefore, smoking cessation in newly diagnosed T2DM patients should be more emphasized to reduce CVD risk and mortality.

Our study has several limitations to be considered when interpreting the results. First, our study population consisted of Koreans, and mostly men, so its generalizability to other ethnicities or women is limited. Our results for women did not show a significant association, probably due to the small number of female smokers. Second, smoking behaviors were assessed based on self-reported questionnaire related to an underestimation of the amount smoked. However, sensitivity and specificity of self-reports of smoking compared to biochemical validation was generally accurate [40]. Third, there could be unmeasured confounding effect such as the exact degree of glycemic control (e.g., glycated hemoglobin), achievement of LDL lowering goal in smokers, and weight gain following smoking cessation. Fourth, we could not assess the exact biological mechanisms regarding the association between smoking behavior change and CVD risk due to a lack of clinical information such as brain imaging.

## Conclusions

In conclusion, smoking cessation was associated with reduced CVD incidence, all-cause mortality, and CVD mortality among T2DM patients consistent with the general population. However, smoking reduction did not mediate such risks. To reduce CVD risk and all-cause mortality, and CVD mortality, smoking cessation should be achieved rather than smoking reduction.

## Electronic supplementary material

Below is the link to the electronic supplementary material.

Supplementary Material 1: **Supplementary table S1** The association of smoking behavior change with myocardial infarction, ischemic stroke, and mortality stratified by previous smoking level. **Supplementary table S2** The association of smoking behavior change with myocardial infarction, ischemic stroke, and mortality stratified by age. **Supplementary table S3** The association of smoking behavior change with myocardial infarction, ischemic stroke, and mortality stratified by sex.

Supplementary Material 2: **Supplementary figure S1** Association of smoking behavior change on the all-cause, myocardial infarction, and ischemic stroke mortality according to diabetes mellitus severity. HR, hazard ratio; CI, confidence interval. HRs were adjusted for age, sex, income, area of residence, alcohol consumption, duration of smoking, physical activity, body mass index, comorbidities (hypertension, dyslipidemia, chronic kidney disease, and chronic obstructive pulmonary disease), fasting glucose, duration of diabetes, and use of insulin.

## Data Availability

The data that support the findings of this study are available from the Korean National Health Insurance Service (KNHIS) and were used under license for the current study (http://nhiss.nhis.or.kr). Restrictions apply to their availability (data are not publicly available). Data are available with permission of the KNHIS from the authors upon reasonable request.
