# Supplementary material for: Smoking behavior change and risk of cardiovascular disease incidence and mortality in patients with type 2 diabetes mellitus
Source: Cardiovasc Diabetol. 2023 Jul 29;22:193. doi: 10.1186/s12933-023-01930-4 (PMC10387213; doi:10.1186/s12933-023-01930-4)
Supplement: Supplementary file 1 — Supplementary Material 1: Supplementary table S1 The association of smoking behavior change with myocardial infarction, ischemic stroke, and mortality stratified by previous smoking level. Supplementary table S2 The association of smoking behavior change with myocardial infarction, ischemic stroke, and mortality stratified by age. Supplementary table S3 The association of smoking behavior change with myocardial infarction, ischemic stroke, and mortality stratified by sex. [file 12933_2023_1930_MOESM1_ESM.docx]

**Supplementary Table S1**. The association of smoking behavior change with myocardial infarction, ischemic stroke, and mortality stratified by previous smoking level

| Smoking behavior change | Light smoker (<10 cigarettes/day) | | | Moderate smoker (10–19 cigarettes/day) | | | Heavy smoker (≥20 cigarettes/day) | | |
| --- | --- | --- | --- | --- | --- | --- | --- | --- | --- |
|  | Events  (n) | IR | aHR^*^ (95% CI) | Events  (n) | IR | aHR^*^ (95% CI) | Events  (n) | IR | aHR^*^ (95% CI) |
| **Cardiovascular disease events** | | | | | | | | | |
| Myocardial infarction (P for interaction 0.779) | | | | | | | | | |
| Quitter | 198 | 3.6 | **0.79 (0.62–1.00)** | 429 | 3.5 | **0.84 (0.75–0.95)** | 544 | 4.0 | **0.81 (0.74–0.89)** |
| Reducer I | 18 | 3.5 | 0.87 (0.53–1.43) | 98 | 3.8 | 0.94 (0.76–1.16) | 457 | 4.6 | 1.04 (0.94–1.15) |
| Reducer II | 45 | 4.0 | 0.92 (0.65–1.31) | 234 | 3.2 | 0.92 (0.79–1.06) | 544 | 3.9 | 1.04 (0.95–1.15) |
| Sustainer | 107 | 4.0 | 1 (Ref.) | 798 | 3.3 | 1 (Ref.) | 1,951 | 3.9 | 1 (Ref.) |
| Increaser | 235 | 4.0 | 1.03 (0.82–1.30) | 578 | 3.7 | **1.14 (1.03–1.27)** | 278 | 4.3 | 1.12 (0.99–1.27) |
| Ischemic stroke (P for interaction 0.861) | | | | | | | | | |
| Quitter | 276 | 5.0 | 0.89 (0.73–1.10) | 513 | 4.2 | **0.83 (0.75–0.93)** | 631 | 4.6 | **0.79 (0.72–0.86)** |
| Reducer I | 22 | 4.3 | 0.80 (0.51–1.25) | 127 | 4.9 | 0.95 (0.79–1.14) | 550 | 5.6 | 1.00 (0.91–1.10) |
| Reducer II | 63 | 5.6 | 0.99 (0.74–1.34) | 302 | 4.1 | 0.95 (0.83–1.08) | 583 | 4.1 | 0.97 (0.88–1.06) |
| Sustainer | 140 | 5.3 | 1 (Ref.) | 970 | 4.0 | 1 (Ref.) | 2,356 | 4.8 | 1 (Ref.) |
| Increaser | 329 | 5.7 | 1.15 (0.95–1.40) | 651 | 4.2 | 1.07 (0.97–1.18) | 324 | 5.1 | 1.12 (1.00–1.26) |
| **Mortality** | | | | | | | | | |
| All-cause mortality (P for interaction 0.566) | | | | | | | | | |
| Quitter | 593 | 10.7 | 0.96 (0.83–1.10) | 1,003 | 8.2 | **0.86 (0.79–0.93)** | 1,285 | 9.3 | **0.92 (0.87–0.98)** |
| Reducer I | 62 | 12.1 | 1.05 (0.80–1.39) | 285 | 11.0 | 1.05 (0.92–1.19) | 1,173 | 11.7 | **1.16 (1.09–1.24)** |
| Reducer II | 151 | 13.3 | 1.07 (0.88–1.30) | 622 | 8.4 | 0.99 (0.91–1.09) | 1,059 | 7.5 | 1.04 (0.97–1.11) |
| Sustainer | 296 | 11.0 | 1 (Ref.) | 1,876 | 7.6 | 1 (Ref.) | 4,122 | 8.2 | 1 (Ref.) |
| Increaser | 637 | 10.8 | 1.06 (0.93–1.22) | 1,232 | 7.9 | **1.08 (1.00–1.16)** | 536 | 8.3 | **1.12 (1.03–1.23)** |
| Myocardial infarction mortality (P for interaction 0.577) | | | | | | | | | |
| Quitter | 27 | 0.5 | 0.70 (0.38–1.29) | 44 | 0.4 | 0.78 (0.54–1.13) | 51 | 0.4 | 0.77 (0.56–1.06) |
| Reducer I | 2 | 0.4 | 0.62 (0.14–2.66) | 17 | 0.7 | 1.43 (0.85–2.41) | 50 | 0.5 | 1.14 (0.83–1.56) |
| Reducer II | 10 | 0.9 | 1.26 (0.58–2.76) | 24 | 0.3 | 0.85 (0.54–1.34) | 42 | 0.3 | 0.92 (0.66–1.29) |
| Sustainer | 17 | 0.6 | 1 (Ref.) | 83 | 0.3 | 1 (Ref.) | 176 | 0.4 | 1 (Ref.) |
| Increaser | 24 | 0.4 | 0.71 (0.38–1.32) | 52 | 0.3 | 1.03 (0.73–1.46) | 28 | 0.4 | 1.31 (0.88–1.96) |
| Ischemic stroke mortality (P for interaction 0.936) | | | | | | | | | |
| Quitter | 12 | 0.2 | 0.56 (0.24–1.31) | 17 | 0.1 | 0.60 (0.34–1.06) | 17 | 0.1 | 0.75 (0.44–1.30) |
| Reducer I | 1 | 0.2 | 0.48 (0.06–3.77) | 6 | 0.2 | 0.79 (0.34–1.88) | 18 | 0.2 | 1.03 (0.61–1.76) |
| Reducer II | 5 | 0.4 | 0.99 (0.34–2.89) | 11 | 0.1 | 0.78 (0.40–1.53) | 15 | 0.1 | 1.12 (0.63–1.97) |
| Sustainer | 10 | 0.4 | 1 (Ref.) | 41 | 0.2 | 1 (Ref.) | 57 | 0.1 | 1 (Ref.) |
| Increaser | 17 | 0.3 | 0.89 (0.41–1.94) | 23 | 0.1 | 1.02 (0.61–1.70) | 3 | 0.0 | 0.53 (0.16–1.68) |

IR, incidence rate per 1,000 person-years; aHR, adjusted hazard ratio; CI, confidence interval.

^*^Adjusted for age, sex, income, area of residence, alcohol consumption, duration of smoking, physical activity, body mass index, comorbidities (hypertension, dyslipidemia, chronic kidney disease, and chronic obstructive pulmonary disease), glucose, duration of diabetes, and use of insulin.

**Supplementary Table S2**. The association of smoking behavior change with myocardial infarction, ischemic stroke, and mortality stratified by age

| Smoking behavior change | Age <65 years | | | Age ≥65 years | | |
| --- | --- | --- | --- | --- | --- | --- |
|  | Events  (n) | IR | aHR^*^ (95% CI) | Events  (n) | IR | aHR^*^ (95% CI) |
| **Cardiovascular disease events** | | | | | | |
| Myocardial infarction (P for interaction 0.712) | | | | | | |
| Quitter | 782 | 3.0 | **0.79 (0.73–0.86)** | 389 | 7.4 | **0.83 (0.73–0.95)** |
| Reducer I | 390 | 3.6 | 1.03 (0.92–1.14) | 183 | 9.1 | 1.06 (0.90–1.24) |
| Reducer II | 653 | 3.2 | 1.01 (0.93–1.11) | 170 | 7.5 | 0.92 (0.78–1.09) |
| Sustainer | 2,233 | 3.2 | 1 (Ref.) | 623 | 8.1 | 1 (Ref.) |
| Increaser | 837 | 3.4 | 1.06 (0.98–1.15) | 254 | 8.7 | 1.05 (0.90–1.21) |
| Ischemic stroke (P for interaction 0.648) | | | | | | |
| Quitter | 801 | 3.1 | **0.77 (0.71–0.84)** | 619 | 12.0 | **0.84 (0.76–0.93)** |
| Reducer I | 413 | 3.8 | 1.00 (0.90–1.11) | 286 | 14.4 | 1.00 (0.88–1.14) |
| Reducer II | 651 | 3.2 | 0.94 (0.87–1.03) | 297 | 13.3 | 0.98 (0.86–1.11) |
| Sustainer | 2,434 | 3.5 | 1 (Ref.) | 1,032 | 13.6 | 1 (Ref.) |
| Increaser | 894 | 3.6 | 1.05 (0.97–1.13) | 410 | 14.2 | 1.02 (0.91–1.14) |
| **Mortality** | | | | | | |
| All-cause mortality (P for interaction 0.036) | | | | | | |
| Quitter | 1,291 | 4.9 | **0.84 (0.79–0.90)** | 1,590 | 30.0 | 0.95 (0.89–1.01) |
| Reducer I | 751 | 6.8 | **1.17 (1.08–1.26)** | 769 | 37.6 | **1.11 (1.03–1.21)** |
| Reducer II | 1,084 | 5.3 | 1.02 (0.95–1.09) | 748 | 32.5 | 1.02 (0.94–1.10) |
| Sustainer | 3,784 | 5.4 | 1 (Ref.) | 2,510 | 32.1 | 1 (Ref.) |
| Increaser | 1,397 | 5.6 | **1.07 (1.00–1.14)** | 1,008 | 33.9 | 1.04 (0.96–1.12) |
| Myocardial infarction mortality (P for interaction 0.993) | | | | | | |
| Quitter | 63 | 0.2 | 0.78 (0.58–1.04) | 59 | 1.1 | 0.81 (0.59–1.12) |
| Reducer I | 35 | 0.3 | 1.11 (0.77–1.60) | 34 | 1.7 | 1.22 (0.83–1.81) |
| Reducer II | 46 | 0.2 | 0.90 (0.65–1.25) | 30 | 1.3 | 1.01 (0.67–1.51) |
| Sustainer | 178 | 0.3 | 1 (Ref.) | 98 | 1.3 | 1 (Ref.) |
| Increaser | 64 | 0.3 | 1.04 (0.78–1.38) | 40 | 1.3 | 1.07 (0.74–1.54) |
| Ischemic stroke mortality (P for interaction 0.321) | | | | | | |
| Quitter | 14 | 0.1 | 0.89 (0.48–1.66) | 32 | 0.6 | **0.58 (0.38–0.88)** |
| Reducer I | 10 | 0.1 | 1.56 (0.77–3.16) | 15 | 0.7 | 0.67 (0.38–1.17) |
| Reducer II | 10 | 0.0 | 1.03 (0.51–2.07) | 21 | 0.9 | 0.95 (0.58–1.54) |
| Sustainer | 35 | 0.1 | 1 (Ref.) | 73 | 0.9 | 1 (Ref.) |
| Increaser | 17 | 0.1 | 1.37 (0.77–2.45) | 26 | 0.9 | 0.85 (0.54–1.32) |

IR, incidence rate per 1,000 person-years; aHR, adjusted hazard ratio; CI, confidence interval.

^*^Adjusted for age, sex, income, area of residence, alcohol consumption, duration of smoking, physical activity, body mass index, comorbidities (hypertension, dyslipidemia, chronic kidney disease, and chronic obstructive pulmonary disease), glucose, duration of diabetes, and use of insulin.

**Supplementary Table S3**. The association of smoking behavior change with myocardial infarction, ischemic stroke, and mortality stratified by sex

| Smoking behavior change | Men | | | Women | | |
| --- | --- | --- | --- | --- | --- | --- |
|  | Events  (n) | IR | aHR^*^ (95% CI) | Events  (n) | IR | aHR^*^ (95% CI) |
| **Cardiovascular disease events** | | | | | | |
| Myocardial infarction (P for interaction 0.126) | | | | | | |
| Quitter | 1,024 | 3.6 | **0.79 (0.73–0.85)** | 147 | 5.5 | 0.92 (0.73–1.17) |
| Reducer I | 533 | 4.4 | 1.05 (0.95–1.15) | 40 | 5.0 | 0.86 (0.61–1.23) |
| Reducer II | 782 | 3.6 | 0.99 (0.91–1.07) | 41 | 5.6 | 1.06 (0.75–1.51) |
| Sustainer | 2,728 | 3.7 | 1 (Ref.) | 128 | 5.4 | 1 (Ref.) |
| Increaser | 1,025 | 3.9 | 1.07 (0.99–1.15) | 66 | 4.8 | 0.88 (0.65–1.18) |
| Ischemic stroke (P for interaction 0.768) | | | | | | |
| Quitter | 1,261 | 4.4 | **0.79 (0.74–0.85)** | 159 | 5.9 | 0.88 (0.70–1.10) |
| Reducer I | 650 | 5.4 | 1.01 (0.92–1.09) | 49 | 6.1 | 0.91 (0.66–1.26) |
| Reducer II | 906 | 4.1 | 0.95 (0.89–1.03) | 42 | 5.8 | 0.96 (0.68–1.35) |
| Sustainer | 3,323 | 4.5 | 1 (Ref.) | 143 | 6.1 | 1 (Ref.) |
| Increaser | 1,209 | 4.6 | 1.03 (0.97–1.10) | 95 | 7.0 | 1.11 (0.85–1.44) |
| **Mortality** | | | | | | |
| All-cause mortality (P for interaction 0.055) | | | | | | |
| Quitter | 2,602 | 9.0 | **0.88 (0.84–0.92)** | 279 | 10.2 | 1.13 (0.94–1.36) |
| Reducer I | 1,431 | 11.6 | 1.14 (1.08–1.21) | 89 | 10.9 | 1.18 (0.92–1.52) |
| Reducer II | 1,766 | 8.0 | 1.02 (0.96–1.07) | 66 | 9.0 | 1.10 (0.83–1.46) |
| Sustainer | 6,104 | 8.1 | 1 (Ref.) | 190 | 8.0 | 1 (Ref.) |
| Increaser | 2,281 | 8.6 | 1.06 (1.01–1.11) | 124 | 9.0 | 1.06 (0.84–1.33) |
| Myocardial infarction mortality (P for interaction 0.595) | | | | | | |
| Quitter | 109 | 0.4 | **0.76 (0.61–0.96)** | 13 | 0.5 | 1.20 (0.50–2.90) |
| Reducer I | 64 | 0.5 | 1.14 (0.87–1.50) | 5 | 0.6 | 1.65 (0.54–5.05) |
| Reducer II | 73 | 0.3 | 0.93 (0.72–1.21) | 3 | 0.4 | 1.18 (0.31–4.46) |
| Sustainer | 268 | 0.4 | 1 (Ref.) | 8 | 0.3 | 1 (Ref.) |
| Increaser | 101 | 0.4 | 1.08 (0.85–1.35) | 3 | 0.2 | 0.63 (0.17–2.36) |
| Ischemic stroke mortality (P for interaction 0.717) | | | | | | |
| Quitter | 42 | 0.1 | **0.68 (0.47–0.98)** | 4 | 0.1 | 0.66 (0.16–2.63) |
| Reducer I | 23 | 0.2 | 0.89 (0.57–1.40) | 2 | 0.2 | 1.06 (0.19–5.78) |
| Reducer II | 29 | 0.1 | 0.96 (0.63–1.45) | 2 | 0.3 | 1.46 (0.27–7.97) |
| Sustainer | 104 | 0.1 | 1 (Ref.) | 4 | 0.2 | 1 (Ref.) |
| Increaser | 37 | 0.1 | 0.94 (0.64–1.37) | 6 | 0.4 | 2.09 (0.59–7.41) |

IR, incidence rate per 1,000 person-years; aHR, adjusted hazard ratio; CI, confidence interval.

^*^Adjusted for age, sex, income, area of residence, alcohol consumption, duration of smoking, physical activity, body mass index, comorbidities (hypertension, dyslipidemia, chronic kidney disease, and chronic obstructive pulmonary disease), glucose, duration of diabetes, and use of insulin.
