# Supplementary figures and images for: Smoking behavior change and risk of cardiovascular disease incidence and mortality in patients with type 2 diabetes mellitus
Source: Cardiovasc Diabetol. 2023 Jul 29;22:193. doi: 10.1186/s12933-023-01930-4 (PMC10387213; doi:10.1186/s12933-023-01930-4)

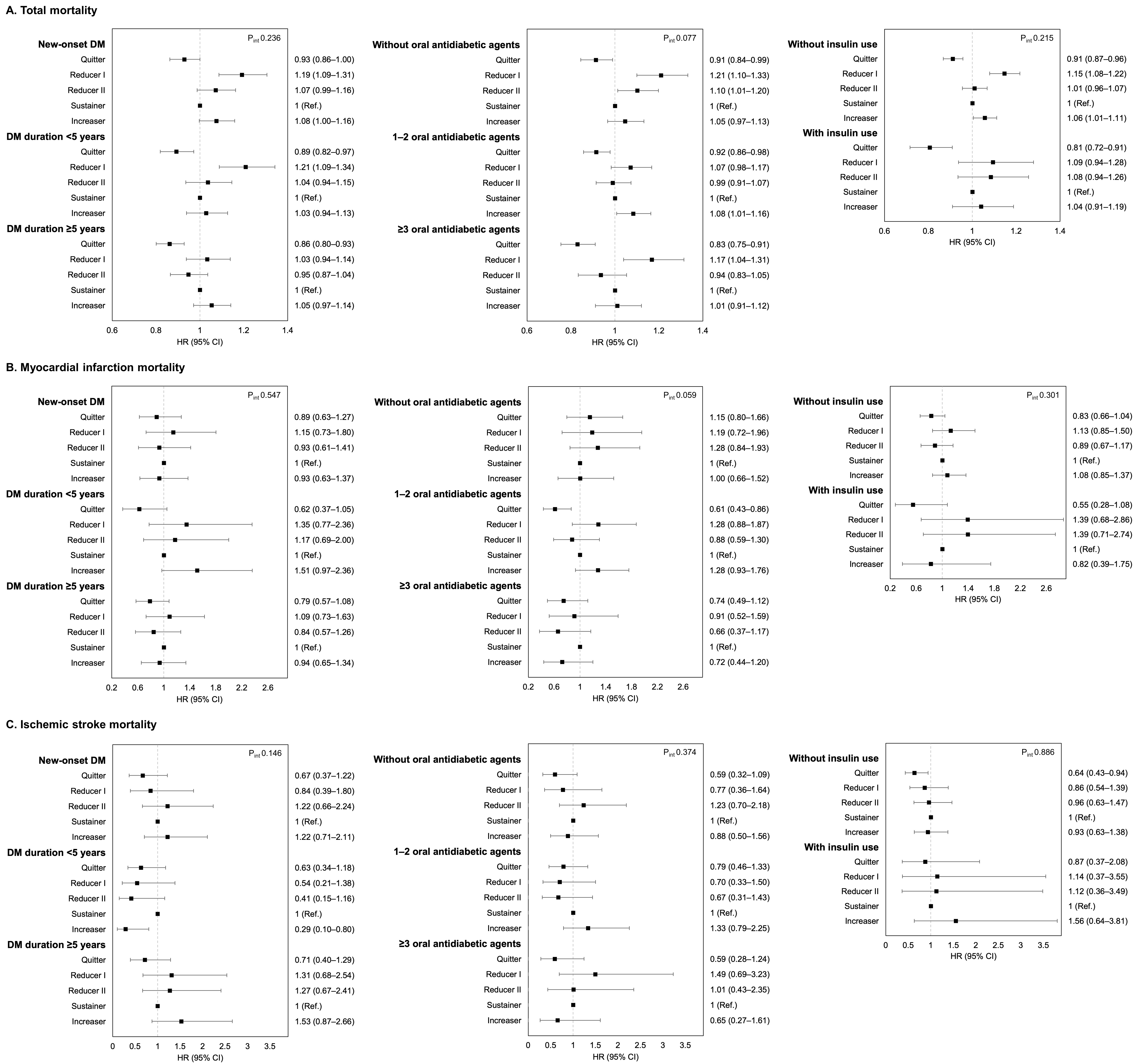

Supplement: Supplementary file 2 — Supplementary Material 2: Supplementary figure S1 Association of smoking behavior change on the all-cause, myocardial infarction, and ischemic stroke mortality according to diabetes mellitus severity. HR, hazard ratio; CI, confidence interval. HRs were adjusted for age, sex, income, area of residence, alcohol consumption, duration of smoking, physical activity, body mass index, comorbidities (hypertension, dyslipidemia, chronic kidney disease, and chronic obstructive pulmonary disease), fasting glucose, duration of diabetes, and use of insulin. [file 12933_2023_1930_MOESM2_ESM.png]
